# The quality of primary care in community health centers: comparison among urban, suburban and rural users in Shanghai, China

**DOI:** 10.1186/s12875-020-01250-6

**Published:** 2020-08-27

**Authors:** Jianwei Shi, Hua Jin, Leiyu Shi, Chen Chen, Xuhua Ge, Yuan Lu, Hanzhi Zhang, Zhaoxin Wang, Dehua Yu

**Affiliations:** 1 School of Public Health, Shanghai Jiaotong University School of Medicine, 227 Chongqing South RD, Shanghai, 200025 China; 2 Department of General Practice, Yangpu Hospital, Tongji University School of Medicine, 450 Tengyue RD, Shanghai, 200090 China; 3 Shanghai General Practice and Community Health Development Research Center, Shanghai, 200090 China; 4 Academic Department of General Practice, Tongji University School of Medicine, 450 Tengyue RD, Shanghai, 200090 China; 5 Department of Health Policy and Management, Primary Care Policy Center, Johns Hopkins University, Baltimore, MD 21205 USA; 6 Shanghai Jing ‘an District Jiangning Road Community Health Service Center, Shanghai, 200041 China

**Keywords:** Primary care, Community health centers, Quality, PCAT

## Abstract

**Objective:**

Following World Health Organization’s initiatives to advance primary care, China put forth forceful policies including the Personal Family Doctor Contract to ensure that every family sign up with a qualified doctor in a community health center (CHC) ever since its 2009 New Health Reform. We used the Johns Hopkins-designed Primary Care Assessment Tool (PCAT) to assess primary care quality experienced by the contracted residents and compare this across different socioeconomic regions.

**Methods:**

Using a multistage sampling method, four CHCs each were randomly selected from urban, suburban and rural districts of Shanghai, a metropolitan with 24 million residents. ANOVA and Multivariate analyses were used to assess the association between location of CHC and the quality of primary care experience.

**Findings:**

A total of 2404 CHC users completed our survey. Except for the domain of coordination (information systems), users from suburban CHCs reported best primary care experiences in all other domains, followed by users of rural CHCs. After controlling for covariates, suburban CHC users were more likely to report higher total PCAT scores (ß = 1.57, *P* <  0.001) compared with those from urban CHCs.

**Conclusion:**

That contracted residents from suburban CHCs reporting better primary care experience than those from urban CHCs demonstrates the unique value of CHCs in relatively medical-underserved areas. In particular, urban CHCs could further strengthen first contact (utilization), first contact (accessibility), coordination (referral system), comprehensiveness (available), and community orientation aspects of primary care performance. However, all CHCs could improve coordination (information system).

## Background

As proposed by World Health Organization (WHO), primary care is a whole-of-society approach that includes health promotion, disease prevention, treatment and rehabilitation, etc. It addresses the majority of a person’s health needs throughout their lifetime, and it is people-centered rather than disease-centered [1]. A strong focus on primary care contributes to the well-functioning of the health care system overall [1, 2]. Previous studies have reported that sound primary care, is well provided by general practitioners in community health institutions in the United States, England, New Zealand, Spain, Canada, etc., [3, 4], helping facilitate health care delivery in these countries. By comparison, China’s primary care system lagged behind and did not receive enough attention until a big shortage of medical resources occurred and led to a lopsided health care delivery system. In 2009, a new round of healthcare reform was launched nationwide in China, in which the government explicitly set a goal to strengthen primary care [5]. Under this reform, 2200 county hospitals and more than 330,000 clinics and rural township hospitals were reconstructed or upgraded into CHCs to ensure that a primary care provider is available to all residents living within a 15-min transportation radius [5].

In 2011, a personal family doctor contract policy was instituted nationwide to encourage residents to utilize services provided by CHCs first when seeking out care. Shanghai, as one of the early cities in China to develop CHCs, put forth specific guidelines to implement the contract in providing comprehensive primary care services, including diagnosis and referral services for common diseases, frequently-occurring disease treatment, chronic disease management, public health services, rehabilitation, nursing and other appropriate community-based medical services [6]. By the end of 2018, there were 6.66 million Shanghai residents (with a sign-up rate of 30%) who participated in the “1 + 1 + 1” (one CHC + one regional secondary hospital + one tertiary hospital) family contract program. The sign-up rate for vulnerable populations such as those 65 and over, pregnant, or disabled, reached 54%. For diabetes and hypertension patients, the rate was over 84% [7]. The reason we only surveyed the contracted residents is that CHCs regard contracted residents as their responsibility or serving as their usual source of care. The PCAT tool explicitly requires the patient’s usual source of care be used to measure his/her primary care experience. Since there is no mandatory restriction on the referral system, any patient in China could bypass CHC and access big hospitals [8]. Thus, regardless of the severity of the illness, many patients are inclined to choose big hospitals because of better medical technology and perceived technical quality, although the expenditure at the hospital setting is much higher than that at the community [8, 9]. In China, to promote the residents to utilize primary care, each contracted resident is assigned a family doctor so that interpersonal relation can be established and care coordination facilitated, both of which critical in patient retention [9].

Heretofore, only a few qualitative case studies and commentaries have been written about the primary care experience of patients seeking CHC care. Hu et al. (2016) evaluated and compared the quality of primary care provided by different types of health care facilities in Guangdong Province of China. And in Wang et al.’s (2013) study, patients aged 18 years or older who visited their health center on the day of recruitment were asked to report the quality of primary care based on a sample of CHCs from Guangdong Province [10, 11]. However, these studies did not explicitly focus on patients who visit health centers as their regular source of care. Strictly speaking, the surveyed respondents may not be in a position to report their primary care experience as captured by PCAT since the tool requires usual source of care as the target. Hence, little is known about the primary care quality experienced by contracted CHC patients and whether there are variations in quality across different socioeconomic regions.

The current study used the primary care assessment tool (PCAT) to examine the quality of primary car experience by CHC users across different socioeconomic regions. Results of the study not only demonstrates the quality of primary care provided by CHCs for their contracted users, but also assesses if there are disparities in primary care performance for contract residents across different socioeconomic regions. Although carried out in China, our study could have implications for other cities or regions undergoing urbanization and reorganizing healthcare delivery and further advance the role of CHCs as a community-based primary care provider.

## Methods

### Study setting

In this study, we chose Shanghai metropolitan because its primary care system is well-developed and represents one of the best in China. At the end of 2019, Shanghai had a population of 14.50 million registered residents and 9.80 million non-registered residents, and its GDP per capita was the highest in China (113.6 thousand RMB) [12]. Shanghai is also often the pilot of national healthcare reform and policy implementation. Its advanced urbanization but diverse socioeconomic development make it a generalizable region to assess primary care performance by CHCs across varying socioeconomic regions.

Due to regional differences in economic and healthcare resources, the primary care in CHCs varies vastly among different socioeconomic regions. In urban region, the dense distribution of secondary and tertiary hospitals makes residents less inclined to choose CHCs due to the convenience of accessing higher-level hospitals and the lack of limits on obtaining specialist services [13]. In suburban region, on the other hand, more new projects are stationed and hence more investments. For example, in the suburban Pudong District of Shanghai, a new health reform initiative was launched in 2014, allowing for construction subsidies and talent recruitment to spur CHC development [14–16]. In rural region, the average number of GPs at each CHC is significantly lower than in urban and suburban areas [17, 18].

### Data collection

**Fig. 1:**
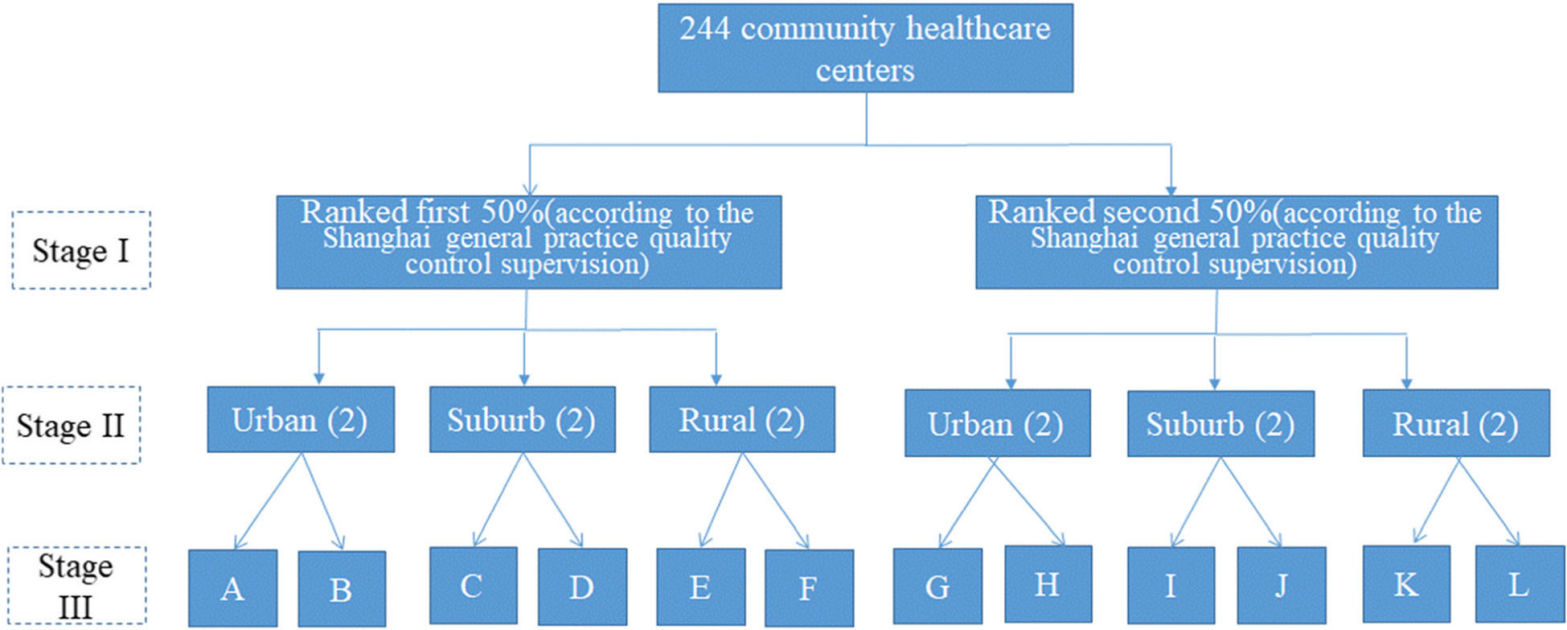
Process of selection of community healthcare centers in various regions in Shanghai

A multistage sampling method was used (Fig. 1). In stage one, we classified all Shanghai CHCs (*n* = 244) into two groups based on their total quality scores as captured by the 2019 Annual Report of Health Center General Practice Quality Performance [19] (i.e., those ranked in the upper 50 percentile and those ranked in the lower 50 percentile) so that both higher and lower performers would be included in the study. In stage two, we classified all CHCs into three clusters based on their geographic location: urban, suburban or rural. Computer-generated random numbers were then used to choose two CHCs from each cluster. In stage three, with the help of local government officials and community residential committees, we contacted the randomly selected CHCs to ask if they would like to participate in our survey. All twelve randomly selected CHCs agreed to participate in our study. The number of patients selected per CHC was calculated by first obtaining the value of proportion of patients who responded favourably to PCAT questions through a pilot (i.e., 85%) and then using 5% as margin of error. This generated a minimum sample size of 200 per CHC which was the sample size we used for selecting patients from the targeted population, i.e., the CHC-contracted residents above 40 years of age. Recruited subjects were selected based on three criteria: 1) aged 40 years or above; 2) were contracted residents in the community, and 3) had visited the given CHC at least twice within the past half year prior to the study. The survey was conducted from August 2019 to December 2019.

### Measurement

Participants’ experiences with primary care were measured using the Primary Care Assessment Tool-Adult Edition (PCAT-AE), which was designed by Professor Barbara Starfield and Leiyu Shi of the Primary Care Policy Center at Johns Hopkins University. It focuses on four exclusive attributes: first contact, longitudinality, comprehensiveness, and coordination. Three supplemental attributes, family centeredness, community orientation, and cultural competence, are also included [20]. Initially applied in the US [20], the PCAT gradually acquired international recognition and has been adapted in other countries with diverse health systems, including Canada [21], Spain [22], Brazil [23], Korea [24] and China [23]. The applications represent the level of primary care provided in various regions and countries and can help by providing specific and targeted directions for improvement [20]. PCAT evaluations have credited the CHC model with providing accessible, cost-effective, and high-quality primary care and reducing health disparities [25, 26]. Its wide adoption across the world makes it a suitable instrument for assessing the quality of primary care in China. In addition, the Johns Hopkins’ team developed a Chinese version and tested it based on adult samples from the southern part of China (Guangzhou Province) and the western part (Tibet Province), the Chinese version of the PCAT questionnaire was proved to have good reliability and validity [27, 28]. In this study, we used the Chinese version of the PCAT validated by the Johns Hopkins team. We obtained the designers’ consent to use the questionnaire for this study. Data were collected through face-to-face interviews and questionnaires were administered by investigators in the cross-sectional study. Since it was used to collect information from adults’ experiences, it was called PCAT-Adult Edition or PCAT-AE. To reduce the presence of interviewer bias, we conducted training with all interviewers prior to actual data collection so that questions and answers were provided consistently. We also conducted a pretest to allow interviewers to practice with actual patients and be monitored. In the early phase of the study, all interviewers were supervised during the actual interview session until they became proficient in administering the questionnaire.

The PCAT-AE was designed to be consistent with the core functions of primary care. A total of 87 items were developed to assess ten domains of participants’ primary care experience: first contact (accessibility and utilization), ongoing care, coordination (information and referral systems), comprehensiveness (service availability and service provided), community orientation, family-centeredness, and cultural competence (Table 1). A four-point Likert-type scale was adopted where 1 = definitely not, 2 = probably not, 3 = probably, 4 = definitely, and 9 = not sure/don’t know (when calculating, 9 was replaced with score of 2.5 based on the PCAT manual). Scores for each domain were derived from the average score of all items within the domain. According to the PCAT Manual, higher scores indicate better patient primary care experience [27, 29].

**Table 1:** Interpretation of PCAT-AE Domains

Domain	Number of items	Interpretation
1. First contact-utilization	3	General routine examination, first diagnosis of new health problems, etc.
2.First contact-accessibility	10	Business hours, receiving medical treatment in one day, telephone consultation, evening home visit, appointment for general physical examination, waiting time, difficulty obtaining medical treatment, expectation value, etc.
3.Ongoing care	14	Receiving care from the same physician/nurse, communication with medical staff, understanding of living and health conditions, etc.
4.Coordination (Referral system)	8	Referral service between primary care and specialists
5.Coordination (Information system)	3	Previous medical records
6.Comprehensiveness (Services available)	32	Available medical services in the CHC
7.Comprehensiveness (Services provided)	6	Some of the services involved in the process
8.Family-centeredness	4	Family involvement in medical procedures, family history
9.Community orientation	5	Family visit, understanding of regional health issues, listening to others
10.Cultural competence	2	Recommended to relatives and friends

In addition, the questionnaire included items about socio-demographic characteristics such as gender, age, marital status, employment status, education, average monthly family income, and health insurance. Items measuring health service utilization were also included, such as the frequency of seeking health services at the CHC, the number of times seeking outpatient service in the past year, self-perceived health status, physical or mental disease lasting over 1 year, and chronic disease.

### Analysis

All data were analysed using SAS Software 9.30. Chi-square tests were conducted to compare socio-demographic characteristics and healthcare utilization of participants among CHCs in the three geographic areas (i.e., urban, suburban, and rural). Analysis of covariance was used to compare PCAT domain scores and total scores among the three types of CHCs. Multivariate linear regression was then performed to explore the relationship between CHC type and reported primary care quality (total PCAT score), controlling for respondents’ socio-demographic and healthcare utilization measures. Two multiple linear regression models were used to explore factors associated with PCAT total scores. Model I included only CHC type, while model II controlled for socio-demographic and healthcare utilization measures. Of all the participants, only 851 contracted residents reported experiencing a referral. Therefore, when conducting the multiple linear regressions, total PCAT scores were calculated by summing the mean scores for all domains except coordination (referral system).

## Results

As shown in Table 2, the proportion of respondents from urban, suburban, and rural areas was roughly similar (31.91, 34.07 and 34.03%, respectively). In total, there were more female (54.78%), 61–70-year-old (47.80%), married (98.88%), and unemployed/retired (63.85%) respondents. Most individuals’ highest education was either primary school or below (37.44%) or junior school (35.27%), and 34.73% had an average monthly family income < 3000 RMB. 82.53% had health insurance. In terms of health service utilization, the majority sought services at CHCs more than once per month (72.80%). A higher proportion sought outpatient services less than 10 times in the previous year (33.32%), followed by > 20 (27.08%) and 10–15 times (26.04%). The majority respondents did not have inpatient hospitalization in the previous year (86.69%). Most respondents reported poor/fair health status (57.45%), and most also reported having no physical or mental disease lasting over 1 year (70.63%). The majority of participants had at least one chronic disease (89.06%).

**Table 2:** Comparison of Participants’ Characteristics from CHCs in Urban, Suburban, and Rural Areas of Shanghai

Variable	Group			District						Chi-square	*P* value
		Total (*n* = 2404)		Urban (*n* = 767)		Suburb (*n* = 819)		Rural (*n* = 818)			
		N	%	N	%	N	%	N	%		
**Socio-demographic characteristics**											
Gender	Male	1087	45.22	329	42.89	358	43.71	400	48.90	6.90	0.03
	Female	1317	54.78	438	57.11	461	56.29	418	51.10		
Age (year)	≤ 60	504	20.97	128	16.69	207	25.27	169	20.66	22.62	< 0.001
	61–70	1149	47.80	380	49.54	392	47.86	377	46.09		
	> 70	751	31.24	259	33.77	220	26.86	272	33.25		
Marital status	Married	2377	98.88	752	98.04	813	99.27	812	99.27	7.03	0.03
	Unmarried	27	1.12	15	1.96	6	0.73	6	0.73		
Employment status	Employed	869	36.15	69	9.00	223	27.23	577	70.54	692.06	< 0.001
	Unemployed/retired	1535	63.85	698	91.00	596	72.77	241	29.46		
Education (missing = 6)	Primary school or below	900	37.53	29	3.78	383	46.76	488	59.66	688.04	< 0.001
	Junior school	848	35.36	329	42.89	287	35.04	232	28.36		
	Senior high school	450	18.77	294	38.33	85	10.38	71	8.68		
	College or above	200	8.34	115	14.99	64	7.81	21	2.57		
Average monthly family income (RMB)	< 3000	835	34.73	35	4.56	186	22.71	614	75.06	967.48	< 0.001
	3000–4000	515	21.42	225	29.34	195	23.81	95	11.61		
	4001–6000	503	20.92	259	33.77	187	22.83	57	6.97		
	≥6000	305	12.69	137	17.86	145	17.70	23	2.81		
	Not sure	246	10.23	111	14.47	106	12.94	29	3.55		
Health insurance	No	420	17.47	143	18.64	115	14.04	162	19.80	10.50	0.01
	Yes	1984	82.53	624	81.36	704	85.96	656	80.20		
**Health service utilization**											
Frequency of seeking health service in CHC	More than once per month	1750	72.80	635	82.79	600	73.26	515	62.96	94.51	< 0.001
	Every one to three months	311	12.94	71	9.26	119	14.53	121	14.79		
	More than every three months	245	10.19	43	5.61	75	9.16	127	15.53		
	Don’t know/Not sure	98	4.08	18	2.35	25	3.05	55	6.72		
Times seeking outpatient service in the previous year	≤10	801	33.32	123	16.04	283	34.55	395	48.29	344.99	< 0.001
	10–14	626	26.04	168	21.90	251	30.65	207	25.31		
	15–20	326	13.56	123	16.04	155	18.93	48	5.87		
	> 20	651	27.08	353	46.02	130	15.87	168	20.54		
Hospitalization in the previous year (missing = 27)	0	2084	87.67	665	86.70	729	89.01	690	84.35	14.8	0.01
	1	231	9.72	86	11.21	61	7.45	84	10.27		
	≥2	62	2.61	13	1.69	18	2.20	31	3.79		
Self-perceived health status	Poor/Fair	1381	57.45	504	65.71	342	41.76	535	65.40	125.02	< 0.001
	Good/Excellent	1023	42.55	263	34.29	477	58.24	283	34.60		
Physical or mental disease lasting over one year	Yes	527	21.92	155	20.21	202	24.66	170	20.78	34.95	< 0.001
	No	1698	70.63	579	75.49	563	68.74	556	67.97		
	Not sure	179	7.45	33	4.30	54	6.59	92	11.25		
Chronic disease	Yes	2141	89.06	705	91.92	716	87.42	720	88.02	9.58	0.01
	No	263	10.94	62	8.08	103	12.58	98	11.98		

Table 2 also compares the socio-demographic characteristics and health service utilization among urban, suburban, and rural CHC users. Similar to the total distribution, participants in each area were more likely to be female, 61–70 years of age, married, unemployed/retired. There were less proportion of participants with an educational attainment at the junior or senior high school or above level in rural area. Also, more rural participants had < 3000 RMB monthly family income and were without health insurance. Regarding healthcare utilization, more urban residents visited CHC more than once per month and had outpatient services > 20 times in the previous year. Urban residents were also more likely to have at least one chronic disease but had no physical or mental disease lasting over 1 year.

CHC users generally reported high quality primary care experience especially in the domain of first-contact (utilization), family centeredness, and comprehensiveness (services provided). Specifically, the scores for first contact (utilization) (mean = 3.68), first contact (accessibility) (mean = 3.06), ongoing care (mean = 3.31), coordination (referral system) (mean = 3.32), comprehensiveness (available) (mean = 3.45), comprehensiveness (provided) (mean = 3.45), community orientation (mean = 3.26), culturally competent (mean = 3.37) were significantly higher for suburban participants (*P* <  0.001). However, coordination (information systems) was perceived highest in urban (mean = 2.82) (*P* <  0.001). When comparing the urban and rural areas, it was found that rural CHCs perceived better in terms of first contact (utilization) (mean = 3.50), first contact (accessibility) (mean = 3.02), coordination (referral system) (mean = 3.25), comprehensiveness (available) (mean = 3.17), and community orientation (mean = 3.10) (Table 3).

**Table 3:** Comparison of Various PCAT Domains among CHCs in Urban, Suburban, and Rural Areas

Domain		District			*P* value
		Urban	Suburb	Rural	
First contact (Utilization)	Mean	3.34^C^	3.68^A^	3.50^B^	< 0.001
	SE	0.62	0.43	0.56	
First contact (Accessibility)	Mean	2.57^B^	3.06^A^	3.02^A^	< 0.001
	SE	0.44	0.34	0.48	
Ongoing care	Mean	3.13^B^	3.31^A^	3.05^C^	< 0.001
	SE	0.39	0.31	0.48	
Coordination (Referral system)	Mean	3.07^B^	3.32^A^	3.25^A^	< 0.001
	SE	0.72	0.49	0.51	
Coordination (Information system)	Mean	2.82^A^	2.68^B^	2.78^A^	< 0.001
	SE	0.35	0.41	0.36	
Comprehensiveness (Available)	Mean	2.97^C^	3.45^A^	3.17^B^	< 0.001
	SE	0.67	0.36	0.55	
Comprehensiveness (Provided)	Mean	3.15^B^	3.45^A^	3.17^B^	< 0.001
	SE	0.56	0.41	0.57	
Family centeredness	Mean	3.35^A^	3.36^A^	3.11^B^	< 0.001
	SE	0.62	0.59	0.66	
Community orientation	Mean	2.96^C^	3.26^A^	3.10^B^	< 0.001
	SE	0.64	0.52	0.61	
Culturally competent	Mean	3.14^B^	3.37^A^	2.99^C^	< 0.001
	SE	0.61	0.63	0.61	
Total- PCAT	Mean	27.42^C^	29.61^A^	27.90^B^	< 0.001
	SE	3.17	2.47	3.49	

Note: Bonferroni t-test was conducted. A indicated the group with the significant highest score, B with the middle, and C with the lowest one under the Bonferroni t-test. When two of the groups were not significantly different, there were only A and B

The multiple linear regression models indicated that geographic area was significantly associated with total PCAT scores in model I (Table 4). After controlling for socio-demographics and health service utilization, participants in suburban CHCs were more likely to report higher total PCAT scores compared to urban participants (ß = 1.57, *P* <  0.001). Respondents who perceived higher total PCAT scores were also more likely to be older in age (61–70 years: ß = -0.60, *P* < 0.001; > 70 years: ß = -0.52, *P* = 0.01). Also, those with a college education or above (ß = 0.81, *P* < 0.001), with an average monthly family income of ≥6000 RMB (ß = -1.24, *P* < 0.001), had > 20 outpatient visits in the previous year (ß = -1.81, *P* < 0.001), and with self-perceived good/excellent health statuses (ß = 0.35, *P* = 0.01) reported significantly lower total PCAT scores.

**Table 4:** Linear Regressions on Total PCAT Scores

Variable	Group	Model I			Model II		
		ß	T value	*P* value	ß	T value	*P* value
District	Urban	Ref.			Ref.		
	Suburban	2.18	14.16	< 0.001	1.57	8.90	< 0.001
	Rural	0.47	3.05	< 0.01	−0.21	−0.93	0.35
**Socio-demographic characteristics**							
Gender	Male				Ref.		
	Female				−0.07	− 0.57	0.57
Age (year)	≤60				Ref.		
	61–70				−0.60	−3.51	< 0.001
	> 70				−0.52	−2.79	0.01
Marital status	Married				Ref.		
	Unmarried				−0.63	−1.11	0.27
Employment status	Employed				Ref.		
	Unemployed/retired				0.18	1.15	0.25
Education	Primary school or below				Ref.		
	Junior school				−0.22	−1.34	0.18
	Senior high school				−0.13	− 0.63	0.53
	College or above				0.81	3.11	< 0.001
Average monthly family income (RMB)	< 3000				Ref.		
	3000 – 4000				−0.07	−0.34	0.73
	4001– 6000				−0.33	−1.62	0.11
	≥6000				−1.24	−5.21	< 0.001
	Not sure				−2.07	−8.46	< 0.001
Health insurance	No				Ref.		
	Yes				0.20	1.25	0.21
**Health service utilization**							
Frequency of seeking health service in CHC	More than once per month				Ref.		
	Every one to three months				−0.66	−3.15	< 0.001
	More than every three months				−0.56	−2.38	0.02
	Don’t know/Not sure				0.02	0.07	0.94
Times seeking outpatient service in the previous year	≤10				Ref.		
	10–15				−0.34	−1.81	0.07
	15–20				0.02	0.10	0.92
	> 20				−1.81	−9.27	< 0.001
Times seeking inpatient service in the previous year	0				Ref.		
	1				0.05	0.27	0.79
	≥2				0.43	1.13	0.26
Self-perceived health status	Poor/Fair				Ref.		
	Good/Excellent				0.35	2.58	0.01
Physical or mental disease lasting over one year	Yes				Ref.		
	No				−0.49	−3.20	< 0.001
	Not sure				−1.48	−5.75	< 0.001
Chronic disease	Yes				Ref.		
	No				− 0.81	−3.70	< 0.001
Adjusted R square			0.086		0.204		

## Discussion

By using the internationally developed and Chinese validated PCAT, we examined contracted residents’ primary care experience in CHCs situated in urban, suburban, and rural areas of Shanghai Metropolitan. Overall, even though respondents in our study generally reported positive experience with their primary care services, it was found that they gave lower PCAT scores than patients from CHCs in the US. This could to some extent be accounted for by the use of different PCAT versions [27, 30]. Nevertheless, the main explanation might be due to China’s still under-developed primary health care system, especially when compared with developed countries. However, in our study, the absolute differences in domains and total PCAT scores for CHCs across different geographic areas were small, which was comparable to a previous study conducted in other regions of China [11]. When comparing with the other China-based studies, the total PCAT score was a little lower than that of a study conducted in the Guangdong Province [8]. This disparity may be caused by sample differences and the survey tool used. Our study focused on contracted residents who more frequently utilized both medical and health management services provided by CHCs whereas previous study included all CHC users regardless of their usual source of care. Also, the study conducted in the Guangdong Province used an abbreviated version of the PCAT (where only 25 items were used to assess the seven domains of primary care), which had significant differences from the PCAT-AE (where 87 items were developed to assess ten domains of participants’ primary care experience) used in our study. The more abundant and competitive medical services provided in larger hospitals in Shanghai may also lead to worse perceptions of primary care at CHCs.

Interestingly, comparing the perceptions of CHCs in various regions within Shanghai indicated that contracted patients at suburban CHCs perceived higher total PCAT scores, followed by patients at urban and rural CHCs. In Shanghai and other regions in China, CHC revenue and expenditure are separate, meaning that CHCs obtain all their subsidies from financial investment. The amount of governmental investment is set by the amount of service provided by the CHC in the previous year [31]. As such, CHC development is largely dependent on regional subsidies and the state of surrounding competitive health institutions. Urban areas of Shanghai contain an abundance of secondary and tertiary hospitals. As no strict referral system exists in China [32], the operation of urban CHCs is largely influenced by fewer financial subsidies that may have an impact on primary care quality. Due to advanced urbanization planning, regional suburban governments obtain more financial investment from the Shanghai municipal government [32]. There is also less competition as fewer large hospitals exist in the suburbs. These added benefits are conducive to CHC development and may improve the quality of primary care in suburban areas. However, comparing rural and urban areas, residents’ perceived PCAT scores were not significantly different, which is not consistent with a previous study conducted in the Guangdong province [33]. This may also be explained by greater competition experienced in urban area (compared to rural area) but less investment received (compared with suburb area) [32].

Regarding the various domains of the PCAT, our results showed that CHCs in suburban districts performed the best in all PCAT domains except for coordination (information systems). This domain represents the convenience of access to patients’ electronic medical records and was found to be best in urban CHCs. This can be explained by the fact that information system development was undertaken by the local urban district for both CHCs and higher-level hospitals. Benefiting from a unified information construction effort, CHCs in urban areas acquired better access to patient medical information [34]. However, among all individual domain scores on the PCAT, the average score for information systems was still the lowest. This indicates that much can be done to improve this specific area. It should be noted that ongoing care/continuity is particularly important for primary care patients, as contracted residents are more likely to use health services more frequently and can benefit from a closer patient-provider relationship [8]. However, CHCs in rural areas have much room for improvement in this domain. Regarding the other domains of first contact (utilization), first contact (accessibility), coordination (referral system), comprehensiveness (available), and community orientation, higher scores were given in rural areas than urban areas. This could possibly be due to the following factors: convenient travel distance to CHCs, no appointments required, and shorter waiting time in rural CHCs [8]. These results differ from an early study based on a sample of 645 adult users from Canada (in Quebec and Nova Scotia), which reported poorer first-contact access in rural areas than in urban areas [35].

Our results indicated that respondents who were older and in relatively good health would perceive higher total PCAT scores. This was consistent with a Korean study based on sample data collected from patients whose usual source of care came from family doctors working at nine private clinics. The Korean Primary Care Assessment Tool also found that primary care quality was positively associated with good self-rated health status [36]. It also found that those with an education of college or above and higher average income would perceive significantly lower total PCAT scores. This may be caused by participants in these groups being more inclined to seek out higher-level hospitals for care. Another previous study in China found that compared with other types of health care facilities, tertiary hospital users had higher proportions of patients with higher education, employment and income levels [8].

Several limitations must be taken into account for this study. First, although the sampling of CHCs was randomly chosen in the cross-sectional study, the sampling of contracted residents was not well-randomized. Participants were selected at each CHC as they were seeking out services, making the age of our sample relatively old. Second, survey data were based entirely off of self-reports and thus may be subject to recall bias. Third, the study examined contracted patients’ subjective experiences of primary care rather than objective health outcomes. Patients’ perceived experiences may vary as a result of their expectations and unique characteristics.

## Conclusion

The finding showed that suburban CHC users reported better total primary care experience than urban CHCs, demonstrating the unique value of CHCs in relatively medical underserved areas. That suburban CHC residents reporting better primary care experience than those from urban CHCs demonstrates the unique value of CHCs in relatively medical-underserved areas. In particular, urban CHCs should strengthen first contact (utilization), first contact (accessibility) and coordination (referral system) aspects of primary care performance. However, all CHCs should improve coordination (information system). To improve residents’ experiences of primary care, relevant policies including a strict referral system to ensure CHCs play a gatekeeping role should be implemented. Adequate funding for CHCs should also be provided, especially for those in urban areas. For CHCs in suburban and rural areas, measures should be used to improve their rudimentary information systems. This study may provide evidence for global countries or regions undergoing urbanization to better improve their primary care quality.

## Data Availability

The datasets generated and/or analysed during the current study are available in the Figshare repository (https://figshare.com/s/f9172352bd91f11bf85f).

## Abbreviations

CHCs: Community healthcare centers
PCAT: Primary care assessment tool
WHO: World health organization
PCAT-AE: Primary care assessment tool-adult edition
